# Unveiling the Potential of Phytocannabinoids: Exploring Marijuana’s Lesser-Known Constituents for Neurological Disorders

**DOI:** 10.3390/biom14101296

**Published:** 2024-10-13

**Authors:** Balapal S. Basavarajappa, Shivakumar Subbanna

**Affiliations:** 1 Center for Dementia Research, Nathan Kline Institute for Psychiatric Research, Orangeburg, NY 10962, USA; subbanna.shivakumar@nki.rfmh.org; 2 Department of Psychiatry, New York University Grossman School of Medicine, New York, NY 10016, USA

**Keywords:** endocannabinoids, neurodegenerative disorders, alcohol-use disorders, substance-use disorders, psychiatric disorders, epilepsy, cannabinoid receptors, Alzheimer’s disease, Parkinson’s disease, Huntington’s disease

## Abstract

Cannabis sativa is known for producing over 120 distinct phytocannabinoids, with Δ^9^-tetrahydrocannabinol (Δ^9^-THC) and cannabidiol (CBD) being the most prominent, primarily in their acidic forms. Beyond Δ^9^-THC and CBD, a wide array of lesser-known phytocannabinoids, along with terpenes, flavonoids, and alkaloids, demonstrate diverse pharmacological activities, interacting with the endocannabinoid system (eCB) and other biological pathways. These compounds, characterized by phenolic structures and hydroxyl groups, possess lipophilic properties, allowing them to cross the blood–brain barrier (BBB) effectively. Notably, their antioxidant, anti-inflammatory, and neuro-modulatory effects position them as promising agents in treating neurodegenerative disorders. While research has extensively examined the neuropsychiatric and neuroprotective effects of Δ^9^-THC, other minor phytocannabinoids remain underexplored. Due to the well-established neuroprotective potential of CBD, there is growing interest in the therapeutic benefits of non-psychotropic minor phytocannabinoids (NMPs) in brain disorders. This review highlights the emerging research on these lesser-known compounds and their neuroprotective potential. It offers insights into their therapeutic applications across various major neurological conditions.

## 1. Introduction

Neurodegenerative disorders (NDs) pose a significant global challenge, contributing extensively to disability and mortality [1,2,3]. The World Health Organization (WHO) projects that by 2040, NDs will be the second-leading cause of death worldwide [4]. These disorders involve the steady loss of neurons, resulting in impaired brain function, the breakdown of neuronal networks, and ultimately leading to disability. Despite substantial research efforts, current treatments primarily focus on managing symptoms, as neuronal degeneration often progresses silently for years before clinical signs such as cognitive, motor, or other neurological symptoms manifest. Cell death, synaptic dysfunction, and behavioral abnormalities underlie most neurodegenerative diseases, such as Alzheimer’s disease (AD), Parkinson’s disease (PD), and Huntington’s disease (HD). In AD, amyloid plaques and tau protein aggregates drive neurodegeneration, while synucleinopathies in PD are marked by Lewy body accumulations [5]. HD, in contrast, is caused by a genetic mutation in the huntingtin gene (Htt) involving expanded CAG repeats [6,7,8]. Other conditions like epilepsy and stroke, though distinct in pathology [9], share overlapping features with NDs, including neuronal damage and behavioral symptoms. Environmental factors, such as substance abuse, can exacerbate these conditions, which often lead to chronic cognitive, emotional, and behavioral impairments. Current therapies for NDs primarily focus on symptom management and slowing disease progression. Medications like cholinesterase inhibitors and NMDA receptor antagonists offer limited cognitive improvement in AD [10,11]. At the same time, levodopa and dopamine agonists alleviate PD motor symptoms but fail to halt disease progression [12]. In HD, tetrabenazine reduces chorea but introduces neuropsychiatric side effects [13,14,15,16]. Although advances in gene therapy, stem cell research, and precision medicine offer hope for future treatments, the urgent need remains for novel therapies that manage symptoms and address underlying disease mechanisms with fewer side effects.

The discovery of cannabinoid receptors sparked interest in the endocannabinoid system (eCB), with endogenous ligands like anandamide (AEA) and 2-arachidonoyl-glycerol (2-AG) [17,18] playing critical roles in CNS functions, including pain regulation, neurogenesis, and immune modulation. Though CB1 receptors are abundant in the brain and mediate the psychoactive effects of THC (for references, see [19]), CB2 receptors, primarily found in immune cells, exhibit minimal psychoactivity [20,21,22]. Both receptor types are now recognized for their broader involvement in neuroprotective and anti-inflammatory processes. In recent years, we and others have reviewed a detailed discussion on the function of the eCB system in health and disease [23,24,25,26,27]. Over 120 phytocannabinoids have been identified in *Cannabis sativa* [28], with Δ^9^-tetrahydrocannabinol (Δ^9^-THC) and cannabidiol (CBD) being the most well-known. While Δ^9^-THC is primarily associated with psychotropic effects, both Δ^9^-THC and Δ^8^-THC [29] have demonstrated therapeutic benefits, including appetite stimulation, analgesia, and antiemetic properties [30,31,32,33]. Non-psychotropic cannabinoids like CBD have gained attention for their anti-inflammatory, anxiolytic, and neuroprotective effects [34]. These compounds are now understood to target multiple receptors and biological pathways beyond the classical CB1 and CB2 receptors. Their phenolic structures endow them with potent antioxidant properties, while their lipophilicity facilitates blood–brain barrier (BBB) penetration [35,36,37], making them attractive candidates for treating neurodegenerative diseases.

This review aims to explore the neuroprotective potential of non-psychotropic minor phytocannabinoids (NMPs)—such as CBD, Δ^9^-tetrahydrocannabivarin (THCV), cannabidivarin (CBDV), and cannabigerol (CBG)—in neurodegenerative conditions. These phytocannabinoids are selected based on their abundance, ease of synthesis, and demonstrated therapeutic potential in other areas, such as cancer and inflammatory disorders. Their structural similarity to CBD, a well-studied neuroprotective agent, suggests they may also hold significant promise for future clinical applications.

## 2. Role of NMPs in Epilepsy

### 2.1. Cannabidiol

**Figure 1:**
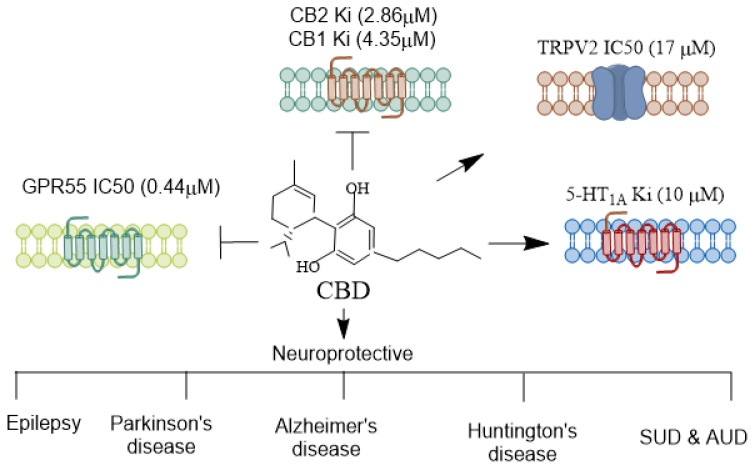
Receptors mediating CBD’s agonistic and antagonistic actions. CBD interacts with various receptors, exhibiting a diverse pharmacological profile. Unlike THC, CBD does not exhibit a high affinity at the CB1 and CB2 receptors but can function as a negative allosteric modulator of CB1 [43], indirectly influencing endocannabinoid signaling. Additionally, CBD acts as an agonist at the 5-HT1A serotonin receptor [44], contributing to its anxiolytic and antidepressant effects. It also modulates TRPV1/2 [45], which are involved in inflammation and pain perception. CBD is an antagonist of GPR55, a receptor implicated in anxiety-related behaviors [46], learning and memory [47], and pain perception [48]. CBD’s receptor specificity contributes to its broad therapeutic potential across various neurological disorders. CBD, Cannabidiol; THC, Δ^9^-tetrahydrocannabinol; TRPV, Transient receptor potential vanilloid. →, agonist; 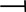, antagonist.

CBD functions as a negative allosteric modulator on CB1 and CB2 receptors (Figure 1). It also exhibits affinity for various other receptors, such as the transient receptor potential vanilloid Type 1 channel (TRPV1), peroxisome proliferator-activated receptor γ (PPARγ), G protein-coupled receptor 55 (GPR55), 5-hydroxytryptamine family 1A (5-HT1A) receptor, γ-aminobutyric acid type A (GABAA) receptor, and transient receptor potential cation channel subfamily M member 8 (TRPM8) (for references, see [19]). However, challenges remain in establishing the neuroprotective function of CBD through any one of these targets. Studies have indicated that eCBs regulate seizure mechanisms in developing animals through CB1 receptor signaling [38]. In pre-clinical seizure models, an anticonvulsant effect was observed due to a pharmacological increase in AEA and 2-AG following neuronal hyperexcitability, which reduces glutamate excitotoxicity during seizures [38,39,40]. Many axon terminals in the CNS express CB1 receptors, which inhibit the release of both excitatory and inhibitory neurotransmitters [41,42].

**Figure 2:**
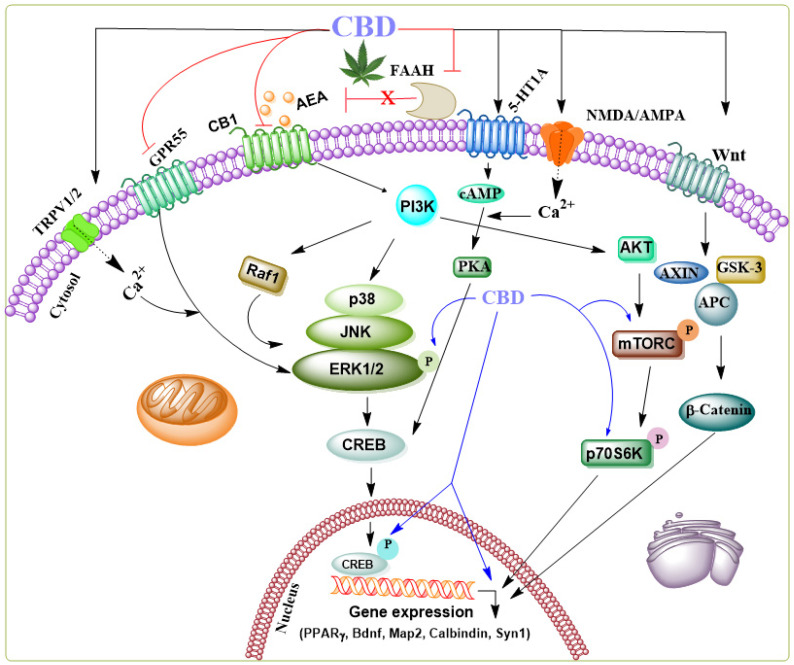
Schematic representation of the signaling pathways modulated by CBD through various receptors in the brain. CBD acts as an agonist, activating several receptors such as 5-HT1A, NMDA/AMPA, Wnt-Frizzled (Fz) family receptors, and TRPV1/2—additionally, CBD functions as a negative allosteric modulator of CB1 and an inhibitor of FAAH, an enzyme involved in the degradation of anandamide (AEA) and an antagonist of GPR55. While the precise mechanisms of action in specific neurodegenerative diseases remain unclear, the collective synergistic effects of CBD have been shown to contribute to its neuroprotective role in several neurological disorder models. CBD’s influence on the ERK1/2 [52], PI3K/AKT [53], and pCREB [60] pathways, which are critical for regulating genes required for cell death, survival, neurogenesis, and synaptic plasticity, including BDNF, MAP-2, synapsin 1, calbindin, and PPARγ, suggests its potential to elicit neuroprotective effects.

Additionally, the anticonvulsant action of CBD involves several mechanisms beyond neuronal death prevention, including reinstating functions of hippocampal interneuron in a temporal lobe epilepsy model [49,50,51]. Furthermore, CBD enhances key molecular components of downstream ERK1/2 [52], PI3K/AKT [53], and pCREB pathways [54] typically linked to endocannabinoid-driven facilitation of adult neurogenesis. Subchronic CBD treatments have been shown to upregulate critical effectors involved in protein synthesis and neuronal survival in the hippocampus, including brain-derived neurotrophic factor (BDNF), MAP-2, calbindin, synapsin 1, and the activation of PPARγ. Further, CBD activates different survival and synaptic remodeling-signaling cascades such as ERK1/2-CREB [55], GSK3β, and PSD95 [56], or PI3K/mTOR/p70S6K [52,57,58,59] (for THE general signaling mechanism see Figure 2).

CBD decreases epileptiform local field potential burst amplitude in the CA1 and dentate gyrus regions in a CB1 receptor-independent manner [61]. This observation is significant because, in addition to CB1 and CB2 receptors, CBD also binds to 5-HT1A receptors in the human brain [62]. These Gi/o protein-coupled serotonergic receptors, when activated, induce inhibitory effects. In temporal lobe epilepsy, 5-HT1A receptor signaling appears crucial in activating seizure-induced cell proliferation and survival in the dentate gyrus [63]. Experimental and clinical findings suggest that serotonergic signaling impacts seizure susceptibility and epilepsy-associated psychiatric comorbidities [64]. A reduction in serotonergic receptor activity in the dentate gyrus may lead to an excitatory/inhibitory imbalance in the trisynaptic circuit, triggering the neuropathology of epilepsy. Another study observed a significant loss of 5-HT1A binding in the hippocampus, temporal cortex, amygdaloid complex, and frontal lobe in patients with temporal lobe epilepsy [65]. Additionally, the downregulation of postsynaptic 5-HT1A receptors exacerbated the severity of epilepsy-associated depression [66]. Decreased serotonin signaling in brain regions related to epilepsy and emotions, such as the hippocampus, amygdaloid complex, and medial prefrontal lobe structures, can promote epilepsy and mental disorders, making CBD a potential therapeutic agent. CBD has also attenuated seizures in genetic models of brainstem and limbic seizures [67]. Thus, several well-designed trials have demonstrated the effectiveness of CBD in controlling epileptic seizures, with minimal significant adverse effects, although agitation, irritability, mood changes, and aggressive behavior have been noted [68]. Based on preclinical and clinical trials, the use of CBD (branded as Epidiolex) for Dravet syndrome, Lennox–Gastaut syndrome, or tuberous sclerosis complex was authorized by the FDA in 2018 and by the European Medicines Agency in 2019.

### 2.2. Δ^9^-Tetrahydrocannabivarin

**Figure 3:**
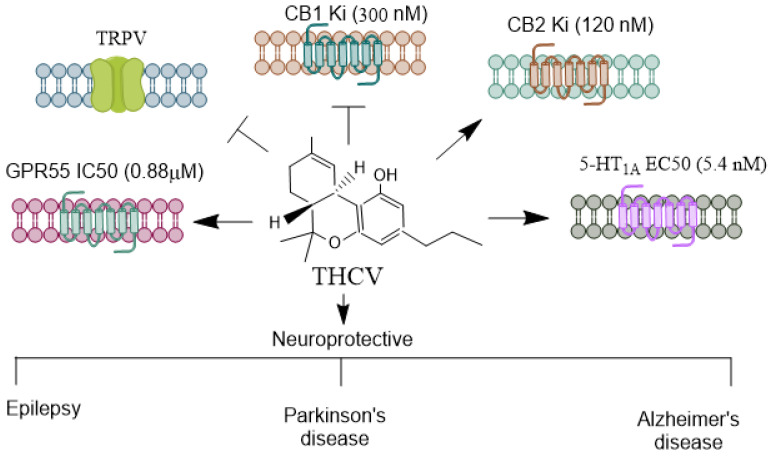
Receptors mediating THCV’s agonistic and antagonistic actions. THCV is a minor cannabinoid with a distinctive pharmacological profile, exhibiting both agonistic and antagonistic effects on cannabinoid and non-cannabinoid receptors. THCV has a unique interaction with CB1 receptors [21,71]. At low doses, it acts as a CB1 antagonist, which can counteract the psychoactive effects of THC. However, at higher doses, THCV can act as a CB1 agonist, producing effects similar to THC, such as appetite suppression and potential psychoactivity. THCV is generally regarded as a partial agonist of CB2, which may contribute to its anti-inflammatory and immunomodulatory properties [21,71]. THCV also modulates transient receptor potential (TRP) channels, particularly TRPV1 [72]. These channels play roles in pain perception, thermoregulation, and inflammation, suggesting THCV’s potential in managing pain and inflammatory conditions. Similar to CBD, THCV has been shown to interact with the 5-HT1A receptor [73], which is involved in spatial learning and memory, anxiety regulation, and mood modulation [74,75]. This receptor interaction may underlie some of THCV’s potential anxiolytic and antipsychotic effects. THCV activates GPR55 [76] anxiety-related behaviors [46], learning, and memory [47]. THCV’s receptor specificity and its ability to shift between agonist and antagonist roles provide it diverse therapeutic potential, particularly in areas like metabolic disorders and neuroprotection. THCV, Δ^9^-Tetrahydrocannabivarin; CBD, Cannabidiol; THC, Δ^9^-tetrahydrocannabinol; TRPV, Transient receptor potential vanilloid. →, agonist; 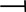, antagonist.

THCV (Figure 3) is a relatively abundant, non-psychoactive phytocannabinoid found in the cannabis sativa [69] plant. It primarily functions as a CB1 receptor antagonist, displacing radiolabeled CB1 agonists in mouse whole-brain membranes [70]. 

THCV also antagonizes CB1-specific GTPγS binding in various mouse brain regions, including the whole brain, cerebellum, and piriform cortex [70,77]. Moreover, it inhibits Δ9-THC-induced anti-nociception and hypothermia in mice [78]. There is evidence from a study in rats that THCV is anti-psychotic in a phencyclidine model of psychosis to a similar degree as clozapine [73]. In tissues enriched with CB2 receptors, THCV acts as a partial antagonist in Gi protein-coupled activities, as evidenced by its effect on forskolin-induced cytosolic cAMP levels [79]. Additionally, THCV regulates calcium transport in epithelial cells by inhibiting TRPV channels [80].

THCV has also demonstrated protective effects in healthy volunteers who exhibited enhanced neural responses to various stimuli [64]. It was shown to be safe, which holds likely for treating central nervous system (CNS) disorders. This makes THCV one of the most promising phytocannabinoids poised for drug approval by regulatory bodies. Indeed, THCV acts as a CB1 receptor antagonist without exerting agonist effects on [³⁵S] GTPγS binding, significantly reducing seizure incidence in adult rat piriform cortex slices [81]. In another study, THCV reduced Purkinje cell firing by enhancing inhibitory neurotransmission at interneuron–Purkinje cell synapses in mouse cerebellar brain slices. This was achieved by decreasing CB1 receptor-mediated, endocannabinoid-induced inhibition of GABA release [82]. The proposed mechanism for THCV’s anticonvulsant action is the increased GABA release through the blockade of CB1 receptors for endocannabinoids [21]. THCV has also shown efficacy in animal models of epilepsy and in NMR neuroimaging studies involving patients with epilepsy [83,84]. However, further studies with varying doses are needed to evaluate its efficacy in epilepsy treatment.

### 2.3. Cannabidivarin

**Figure 4:**
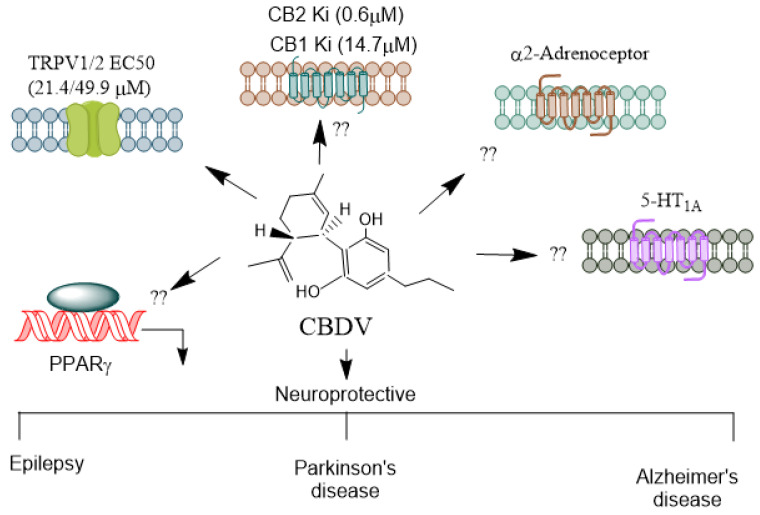
Receptors mediating CBDV’s agonistic and antagonistic actions. CBDV, a non-psychoactive cannabinoid, exhibits both agonistic and antagonistic activity on several receptors, contributing to its potential therapeutic benefits in conditions like epilepsy, developmental disorders, and inflammation. Unlike THC, CBDV has a low affinity for CB1 and CB2 receptors [86]. It is thought to act more as a modulator of the endocannabinoid system rather than directly engaging as an agonist or antagonist at these receptors. This indirect modulation could influence the signaling pathways mediated by endocannabinoids, contributing to its anticonvulsant properties [92]. CBDV significantly affects TRPV1 and TRPV2, where it acts as an agonist [72]. These channels regulate pain, inflammation, and sensory perception, making CBDV potentially useful in managing neuropathic pain and inflammatory conditions. It is unknown whether CBDV activates other receptors such as α2-adrenoreceptors, PPARs, and 5-HT_1A_. CBDV’s receptor specificity highlights its therapeutic potential, particularly in epilepsy, neuroinflammation, and pain management, making it a promising candidate for further medical research. CBD, Cannabidiol; CBDV, Cannabidivarin; THC, Δ^9^-tetrahydrocannabinol; TRPV, Transient receptor potential vanilloid; PPARs, Peroxisome proliferator-activated receptor. →, agonis.

CBDV (Figure 4) is found in unhybridized pure sativa and indica cannabis plants, known as landrace strains. These strains contain high amounts of CBD and low amounts of Δ^9^-THC. CBDV exhibits low binding affinity for the CB1 and CB2 receptors [85,86] and, therefore, appears to lack the psychotropic effects seen with THC. Consistent with this function, high concentrations of CBDV are required to stimulate CB1 receptor-coupled activation of [^35^S] GTPγS binding, inhibit cAMP synthesis, and recruit β-arrestin 2 (βarr2) [85,87,88]. Primarily, CBDV is a more potent and efficacious agonist at CB2 receptors [87,88]. It also activates TRPV1-4 channels [89,90] and stimulates ERK1/2 phosphorylation, inhibiting LPS-mediated signaling [76] through the GPR55 receptor. Additionally, CBDV acts as an inverse agonist at the Gs-coupled GPR6 receptor, leading to the stimulation of adenylyl cyclase and the recruitment of β-arrestin 2 [91]. CBDV has poor oral bioavailability. However, its liposoluble properties enable it to cross the blood–brain barrier [78] efficiently.

CBDV exhibited an antiseizure effect in animal models and a favorable safety profile in human studies [93,94]. In *MECP2*-308 mouse models, CBDV improved sociability, brain weight, and overall health and partially improved motor function [95,96]. In rat studies, CBDV significantly reduced pentylenetetrazol (PTZ) seizure severity and mortality [93,97]. However, CBDV did not affect the severity of pilocarpine convulsions. Further, CBDV significantly suppressed PTZ seizures, decreased seizure severity, and rescued PTZ-induced gene expression [98]. In another study, long-term CBDV administration in RTT mice significantly improved brain weight without affecting the neurotrophin levels [95]. None of these in vivo studies used antagonist experiments further to elucidate the mechanism of CBDV’s anticonvulsant effects. In an adult focal epilepsy cohort, CBDV exhibited safety in humans and significantly reduced seizure frequency [99]. In a pediatric RTT cohort, CBDV significantly improved seizure control in children with *MECP2*-related RTT [100]. Future studies are warranted to establish the specific mechanism CBDV offers for neuroprotective effects.

### 2.4. Cannabigerol

**Figure 5:**
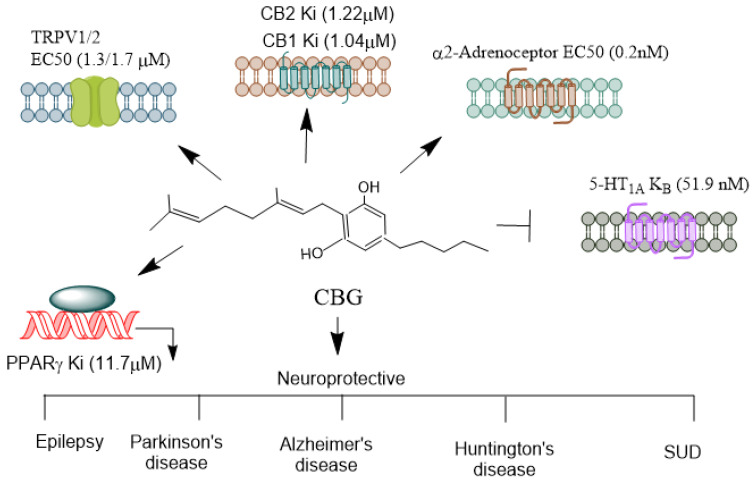
Receptors mediating CBG’s agonistic and antagonistic actions. CBG, often referred to as the “mother cannabinoid” due to its role as a precursor to other cannabinoids like THC and CBD, exhibits a diverse pharmacological profile, interacting with various receptors and modulating multiple physiological pathways. CBG is considered a partial agonist at both CB1 and CB2 receptors [101]. However, its affinity for these receptors is relatively low compared to THC. Its interaction with CB1 is thought to be non-psychoactive and may even counteract some of the psychoactive effects of THC, which acts as a full agonist. CBG’s CB2 agonism has been linked to immunomodulatory and anti-inflammatory effects, which may aid in its therapeutic potential in conditions like neuroinflammation. CBG acts as an agonist at particularly TRPV1/2 [90], which is involved in pain, inflammation, and thermoregulation. CBG can activate alpha (2)-adrenoceptors, and block 5-HT1A receptors [103]. CBG activates PPARγ [102], which are involved in lipid metabolism, inflammation, and cell differentiation. CBG’s receptor interactions highlight its diverse pharmacological profile, making it a likely candidate for therapeutic applications in pain management, neuroprotection, mood disorders, and inflammation. Its ability to act as both an agonist and antagonist across different receptor systems contributes to its potential to treat a wide range of medical conditions. CBG, Cannabigerol; THC, Δ^9^-tetrahydrocannabinol; TRPV, Transient receptor potential vanilloid; PPARγ, Peroxisome proliferator-activated receptor-γ. →, agonist; 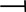, antagonist.

CBG (Figure 5) activates a2-adrenoceptors [101], PPARγ [102], which binds to cannabinoid CB1 and CB2 receptors [101] and blocks CB1 and 5-HT1A receptors [103], and it has been shown to inhibit sodium channel currents in vitro. However, it was ineffective as an anticonvulsant in a PTZ-induced acute seizure model [104]. Furthermore, CBG did not prevent hyperthermia-induced seizures in a Scn1a+/− mouse model of Dravet syndrome, which involves mutations affecting Na_V_ channels [105]. In stably transfected HEK cells and primary dorsal root ganglion (DRG) neurons expressing NaV channels, CBG acted as a low-affinity inhibitor of sodium channels [106]. Another study found that CBG produced modest inhibitory effects on peak currents elicited by this subset of sodium channels in human voltage-gated sodium channel subtypes expressed in mammalian cells [107]. These findings suggest that CBG may induce neuronal hyperexcitability. However, more in vivo studies are warranted to explore its neuroprotective mechanisms in controlling epileptic seizures. The neuroprotective functions of NMPs in epilepsy models are summarized in Table 1.

## 3. Role of NMPs in Parkinson’s Disease

Parkinson’s disease (PD) is the second-most-dominant neurodegenerative condition, affecting approximately 5% of individuals over the age of 85. Recently, the global burden of PD has doubled, making it one of the fastest-growing neurodegenerative diseases. Both genetic and environmental factors contribute to the risk of developing PD [110]. The PD is characterized by motor symptoms such as tremors, rigidity, bradykinesia, and a range of non-motor symptoms [111]. Motor symptoms are strongly associated with the damage and dysfunction of dopaminergic neurons in the nigrostriatal pathway, particularly during the intermediate stages of the disease [112]. This damage is often linked to the presence of α-synuclein, a protein involved in synaptic vesicle release, which can misfold and form aggregates known as Lewy bodies [113]. Developing α-synuclein aggregates can take more than 20 years, indicating that other neurotransmission systems may contribute to early non-motor symptoms, such as mild olfactory and cognitive impairments and depression [111,114]. eCBs, acting via CB1 and CB2 receptors, regulate synaptic and motor functions and can impact striatal rearrangement following dopamine depletion [115,116,117]. In PD subjects, cerebrospinal fluid exhibits heightened levels of AEA [117], and a reduction in CB1 receptors is observed in the substantia nigra [118]. Conversely, increased CB1 receptor expression is found in the nigrostriatal, mesolimbic, and mesocortical dopaminergic projection areas [118]. CB1 receptors also appear to be involved in the action of 3,4-dihydroxy-L-phenylalanine (L-DOPA); CB1 agonists have been shown to prevent the motor fluctuations commonly observed during L-DOPA therapy [119]. Additionally, increased CB2 receptor expression has been found in glial cells in postmortem tissues of PD subjects [120], suggesting a role in the inflammatory aspects of the disease. Due to the role of the eCB system in PD, pharmacological manipulation of the eCB system may represent a potential therapeutic approach for managing the disease.

### 3.1. Cannabidiol

A recent meta-analysis suggested that treatment with CBD significantly improves PD symptoms [121,122]. Nabilone has also been shown to be effective in alleviating anxious moods and night-time sleep problems [123], which are non-motor symptoms of PD. Additionally, CBD exhibits neuroprotective effects on the nigrostriatal pathway [124,125], and in vitro studies indicate its ability to activate tropomyosin receptor kinase A [126]. The anti-apoptotic effects of CBD are mediated by the ERK and Akt/mTOR pathways, with ERK activation modulated by TRPV1 and CB2 receptors [53]. Further studies have demonstrated that CBD exerts anti-apoptotic effects on dopaminergic neurons and reduces neuroinflammation by inhibiting the expression of NLRP3/caspase-1/IL-1β, Bax, and caspase-3 while upregulating Bcl-2 [127]. Therefore, the neuroprotective effects of CBD in PD appear to be mediated by CB2, but not CB1, receptors [53,124]. CBD prevented memory impairments and decreased despair-like behavior that was induced by bilateral intraoral 6-OHDA lesions [128]. In addition, CBD prevented dopaminergic neuronal loss in the striatum, ventral tegmental area, substantia nigra pars compacta (SNpc) and a reduced hippocampal neurogenesis, reduced the mortality rate, and decreased neuroinflammation in 6-OHDA-lesioned rats [128]. Repeated treatment with CBD favored the neuronal maturation of newborn neurons in the hippocampus in Parkinsonian rats [128]. Although these studies provide evidence of the therapeutic potential of CBD, further research is warranted before considering CBD for the treatment of PD.

### 3.2. Δ^9^-Tetrahydrocannabivarin

THCV attenuated the motor impairments induced by 6-hydroxydopamine, likely through changes in glutamatergic transmission [109]. Also, chronic administration of THCV rescued the loss of tyrosine hydroxylase-positive neurons caused by 6-hydroxydopamine in the substantia nigra as antioxidant effects [109]. Additionally, THCV was protective against 6-hydroxydopamine (6-OHDA) or LPS-induced motor impairments similar to rimonabent in rats [109]. Chronic THCV impaired microglial activation and preserved nigrostriatal dopaminergic neurons after 6-OHDA administration and in the LPS model of PD. Additionally, THCV preserved TH-positive neurons, possibly through CB2 receptors [109]. Also, THCV antagonized the effects of the CB1 receptor agonist, CP55,940, indicating its effects as an antagonist of the CB1 receptor. In Pitx3ak mutant mice, THCV rescued abnormal involuntary movements and reduced the FosB protein and the histone pAcH3, which had previously been found to be enhanced in the basal ganglia in L-DOPA-induced dyskinesia [129]. While future investigations are warranted to examine the clinical significance of THCV in humans, the findings establish THCV as a promising agent for developing a nonpsychotrophic cannabinoid-based therapy for patients with PD.

**Table 2:** Neuroprotective functions of NMPs in PD models.

Model	NMPs	Effect	Reference
PD Patients	CBD	PD symptoms ↓	[121]
PD Patients	CBD	Anxiety, tremor amplitude ↓	[122]
Unilateral lesions rat model	CBD	Hydroxydopamine-induced DA depletion	[124]
Sprague–Dawley rats	CBD	Neuroprotection ↑	[125]
PC12 cells	CBD	Cell viability, differentiation, axonal (GAP-43), synaptophysin, and Synapsin I ↑	[126]
SH-SY5Y cells	CBD	Cell viability ↑ Apoptosis, bax, and caspase 3. Moreover, nuclear PARP-1 ↓	[53]
Mice	CBD	Cognitive dysfunction, TNF-α, IL-1β, IL-6, bax, caspase-3, and NLRP3/caspase-1/IL-1β inflammasome pathway ↓ Locomotion, 5-HT, DA, IL-10, TH, and Bcl-2 ↑	[127]
Rats	CBD	SNpc, mortality rate, hippocampal neurogenesis, despair- behavior, memory impairments, and neuroinflammation ↓ Neuronal maturation ↑	[128]
Mice	CBG	Motor tests, LAMP-1, TNF-α, IL-1β, nitric oxide synthase, and COX2 ↓	[130]
SH-SY5Y cells Mice (unilaterally lesioned)	CBG	Cytoprotection, GFAP, and CD68 ↓ Motor activity ↑	[131]
SH-SY5Y cells Mice (unilaterally lesioned	CBG	TH-positive neurons, motor activity ↑	[132]
Rat	Δ^9^-THCV	Motor activity, TH-positive neurons ↑	[109]
Pitx3^ak^ mutant mice		AIMs, horizontal and vertical activities, FosB, pAcH3, and dyskinesia ↓	[129]
*C. elegans*	CBDV	α-syn, DAergic neurons ↓	[133]

CBD, Cannabidiol; CBDV, Cannabidivarin; DA, Dopamine; CBG, Cannabigerol; TH, Tyrosine hydroxylase; GFAP, Glial fibrillary acidic protein; Δ^9^-THCV, Δ^9^-tetrahydrocannabivarin; SH-SY5Y is a thrice-subcloned cell line derived from the SK-N-SH neuroblastoma cell line; *C. elegans*, Caenorhabditis elegans; FosB, FosB Proto-Oncogene, AP-1 Transcription Factor Subunit; pAcH3 phosphoacetyl H3; CD68, Cluster of Differentiation 68; LAMP-1, Lysosome-associated membrane glycoprotein 1; TNF-α, Tumor necrosis factor alpha; IL-1β, Interleukin 1 Beta; IL-10; Interleukin 10; IL-6, Interleukin 6; COX2, Cyclooxygenase 2; Bax, Bcl-2-associated X protein; NLRP3, Nod-like receptor protein 3; 5-HT, 5-hydroxytryptamine; Bcl-2, B-cell lymphoma-2, PARP-1, Poly (ADP-ribose) polymerase 1; SNpc, Substantia Nigra Pars Compacta; GAP-43, Growth-associated protein 43. Pitx3^ak^ mice, Microphthalmia and aphakia mice; PC12 cells, Rat adrenal tumor cells; ↓, reduced; ↑, enhanced.

### 3.3. Cannabidivarin

CBDV has been shown to block α-synuclein (α-syn) aggregation via DAF-16, a key transcription factor involved in regulating oxidative stress, in a transgenic *Caenorhabditis elegans* (*C. elegans*) model [133]. Additionally, CBDV protects dopaminergic neurons from injury and degeneration induced by 6-OHDA [133]. Further exploring the molecular targets through which CBDV ameliorates α-synuclein aggregation in vivo would be valuable for understanding its therapeutic potential in PD. CBDV blocked α-synuclein (α-syn) aggregation via DAF-16, a key transcription factor for regulating oxidative stress in the transgenetic *C. elegans* model [133]. CBDV prevented dopaminergic neurons from 6-OHDA-induced injury and degeneration [133]. The molecular target (s) for CBDV ameliorating α-synuclein aggregation in vivo would be deserving of exploration in the future to understand its therapeutic potential of PD.

### 3.4. Cannabigerol

CBG has recently been investigated for its neuroprotective properties in inflammatory models of PD in mice. In one study, CBG improved motor function and rescued the loss of tyrosine hydroxylase-containing nigrostriatal neurons in mice that were unilaterally lesioned with lipopolysaccharide (LPS) [130]. Additionally, CBG reduced the elevated levels of LAMP-1 immunolabeling (a marker for autophagy impairment) and proinflammatory mediators such as tumor necrosis factor-α, interleukin-1β, inducible nitric oxide synthase and cyclooxygenase-2, and glial reactivity caused by the LPS lesion [130]. In another study using a neurotoxin model of PD, CBG exhibited cytoprotective effects in cultured SH-SY5Y cells treated with 6-hydroxydopamine (6-OHDA) [131]. Additionally, in mice unilaterally lesioned with 6-OHDA, CBG rescued tyrosine hydroxylase (TH)-positive nigral neurons from 6-OHDA-induced damage. It also completely prevented astroglial (GFAP) and microglial (CD68) reactivity in the substantia nigra of lesioned mice, leading to recovery from the motor deficiencies caused by 6-OHDA [130,132]. Furthermore, CBG treatment restored dopamine levels and its metabolite DOPAC in the striatum of 6-OHDA-lesioned mice [131]. These neuroprotective effects were attributed to CBG’s activity at the peroxisome proliferator-activated receptor-γ (PPAR-γ) rather than the CB2 [132]. The neuroprotective functions of NMPs in PD models are summarized in Table 2.

## 4. Role of NMPs in Alzheimer’s Disease

Alzheimer’s disease (AD) is a chronic neurodegenerative condition that affects the central nervous system, leading to a decline in cognitive functions. Similar to other neurodegenerative diseases, AD involves multiple pathologies influenced by a combination of genetic and environmental factors. Several potential causes are associated with the onset of AD, including the deposition of beta-amyloid (Aβ) aggregates forming senile plaques, hyperphosphorylation of Tau protein resulting in neurofibrillary tangles (NFTs), oxidative stress, neuroinflammation due to microglial activation, metabolic dysfunction, and obesity (for references, see [134]).

Despite numerous pharmacological strategies designed to slow down AD symptoms, these approaches have largely been ineffective. In AD patients, senile plaques have been shown to express CB1 and CB2 receptors along with microglial activation markers [135]. However, the number of CB1-positive neurons abundant in healthy individuals is significantly reduced in areas with activated microglia. Additionally, G protein coupling and CB1 receptor protein expression are markedly diminished in AD brains [135]. Furthermore, enhanced CB2 levels are found in AD patients and correlated with brain Aβ42 levels and senile plaque score [136]. The marked eCBs changes, as found in AD patients, were also observed in several AD mouse models [137,138,139]. These findings indicate that cannabinoid receptors play a vital role in the development of AD pathology and suggest that cannabinoids lacking psychiatric effects may have the potential to prevent the neurodegenerative process in AD.

### 4.1. Cannabidiol

In pre-clinical studies, CBD exhibited neuroprotection against many aspects of AD pathology. For example, the application of CBD prevented Aβ-peptide toxicity in PC12 cells by inhibiting oxidative stress, caspase-3 activation [140], and Tau hyperphosphorylation [141]. Additionally, CBD downregulated p-GSK-3β, an inhibitor of the Wnt pathway, causing enhanced Wnt/β-catenin signaling and stimulation of PPARγ and amyloid precursor protein (APP) ubiquitination. In the brain, Wnt/β-catenin signaling is crucial for neuronal survival and neurogenesis. It regulates synaptic plasticity and blood–brain barrier integrity and function [142]. Furthermore, CBD attenuated oxidative stress and rescued mitochondrial function [141]. In mesenchymal stem cells, CBD exposure reduced genes coding for the kinases responsible for Tau phosphorylation and the secretase enzymes involved in Aβ production [143]. Studies have shown that CBD rescues the formation of NFTs and prevents neuronal apoptosis via functioning as an endogenous cannabinoid receptor agonist [144].

In adult mice, intraventricular (i.v.) injection of fibrillar Aβ caused IL-6 gene expression and spatial memory deficits, which were rescued by CBD injection for 2 weeks [145]. In APP × PS1 mice, CBD administration rescued cognitive deficits measured by object recognition and social recognition memory without affecting anxiety behavior [146]. In a similar study, CBD was provided as pellets to APP × PS1 mice at the age of 2.5 months for 8 months and could prevent the development of social recognition memory deficits without affecting Aβ load, oxidative damage, or anxiety behavior [147]. In 5xFAD mice, chronic, low-dose CBD administration significantly improved memory and enhanced an insoluble form of Aβ levels in the cortical regions [148]. Oral administration of CBD for an extended period reversed pathophysiology and rescued social recognition memory deficits in the APP × PS1 mouse model for AD [149]. CBD administration in the senescence-accelerated mouse prone 8 (SAMP8) model improved cognitive function, increased hippocampal-activated microglia shift from M1 to M2, and restored gut microbial functions [150]. Additionally, CBD stimulates autophagy signal transduction through crosstalk between ERK1/2 and AKT kinases [52], which are key cell proliferation and survival regulators and have been implicated in AD pathology. In a randomized, double-blinded, placebo-controlled trial, CBD improved behavioral symptoms in AD [151]. Future studies are warranted to address research issues related to CBD’s safety, efficacy, and variability. In this direction, nanoparticle-coated CBD exhibited better beneficial effects on rescuing learning and memory, increasing hippocampal CB1 and CB2 receptors while reducing amyloid plaques in an AD rat model [152]. Additionally, administering CBD along with THC, a combination known to provide more significant therapeutic benefits than phytocannabinoids alone [153] was shown to improve behavior problems and rigidity in severely demented patients [154].

**Table 3:** Neuroprotective role of NMPs in AD models.

Model	NMPs	Effect	Reference
PC12 cells	CBD	Cell Survival ↑ ROS, lipid peroxidation, and caspase 3 ↓	[140]
PC12 cells	CBD	Wnt/β-catenin ↑ Tau hyperphosphorylation and p-GSK-3β ↓	[141]
MSC cells	CBD	*GSK3*β, *CDK5*, *DYRK1A*, *CAMK2A*, *MAPK1*, *MAPK12*, *MAPK14,* and *BACE1* ↓,	[143]
N13 microglial cells Rat primary microglia Mice	CBD	Intracellular calcium ↓ Nitrite generation and IL-6 gene expression ↓ Spatial memory and microglial migration ↑	[145]
APPxPS1 mice	CBD	Social recognition and novel object recognition ↑	[146]
AβPPSwe/PS1ΔE9	CBD	Social recognition ↑	[147]
5x FAD mice	CBD and THC	Spatial memory and beta amyloid ↑	[148]
SAMP8 mice	CBD	Bacteriodetes and hippocampal-activated microglia shift from M1 to M2 ↑ and LPS ↓	[150]
Male wistar rats	CBD coated by nano-chitosan	Learning and memory and CB1 and CB2 protein expression ↑ Amyloid plaques ↑	[152]
Female patients	CBD	Direct contact and behavior ↑	[154]
MC65 cell	CBG	Aβ aggregation ↓	[155]
PC12 cells	CBG	Aβ aggregation and Aβ_1-42_ neurotoxicity ↓ Neuroprotection	[156]
Male human subjects	∆^9^-THCV	Memory impairment ↓	[157]
In vitro assay	CBD, CBDV, CBG	AChE and BChE ↓	[158]

CBD, Cannabidiol; CBDV, Cannabidivarin; THC, Tetrahydrocannabinol; CBG, Cannabigerol; ∆^9^-THCV, ∆^9^-tetrahydrocannabivarin; ROS, Reactive oxygen species, MSCs, Mesenchymal stem cells; LPS, Lipopolysaccharide; AChE, Acetylcholinesterase; BChE, Butyrylcholinesteras; BDNF, Brain-derived neurotrophic factor; PGC1α, Peroxisome proliferator-activated receptor gamma coactivator 1-alpha; CDK5, Cyclin-dependent kinases 5 gene; DYRK1A, Dual-specificity tyrosine phosphorylation-regulated linase 1A; CAMK2A, Calcium-/calmpdulin-dependent protein kinase II α; MAPK1, Mitogen-activated protein kinase 1; MAPK12, Mitogen-activated protein kinase 12; MAPK14, Mitogen-activated protein kinase 14; BACE, β-Secretase 1; PC12 cells, Rat adrenal tumor cells; MSC, Multipotent stromal cells; N13 cells and primary mice microglial cells; APP, Amyloid precursor protein; PS1, Presenilin 1; FAD, 5 familial AD; SAMP8, Senescence accelerated mouse prone 8; MC65, Human CNS nerve cell line; HT22, Mouse hippocampal Neuron cell; ↓, reduced; ↑, enhanced.

### 4.2. Δ^9^-Tetrahydrocannabivarin

While THCV has not yet been tested in AD models, it has demonstrated a strong safety profile in healthy adults. In a placebo-controlled, double-blind, crossover pilot study, THCV was shown to reverse THC-induced memory impairment [157]. Future studies using THCV in AD animal models could provide potential neuroprotective mechanisms and therapeutic benefits.

### 4.3. Cannabidivarin

CBDV has demonstrated efficacy in an in vitro AD model by blocking oxytosis and energy loss in HT22 cells and reducing Aβ toxicity and trophic withdrawal. However, these experiments did not investigate the specific mechanisms underlying these effects [155]. Additionally, CBDV was found to inhibit the activities of acetylcholinesterase (AChE) and butyrylcholinesterase (BChE) [158]. Abnormal hyperactivity of AChE and BChE contributes to cholinergic deficiency, associated with several neurological disorders, including the memory impairments and cognitive decline observed in AD [159]. Therefore, further research to elucidate the molecular mechanisms of CBDV in AD models could provide valuable insights into its therapeutic potential for this condition.

### 4.4. Cannabigerol

CBG has been shown to reduce the accumulation of Aβ deposits in vitro. Specifically, CBG cleared the preformed and aggregated Aβ from neurons and stimulated Aβ degradation in the MC65 cell line [155]. This widely recognized human neuron-like cell line lacks CB1 or CB2 receptors extensively used for proteotoxicity studies [155]. Additionally, CBG inhibited Aβ_1-42_ -induced neurotoxicity and morphological changes such as reduced neuritic projections and rounded cell morphology in PC12 cells [156]. Future studies with CBG in AD animal models could help elucidate this phytocannabinoid’s neuroprotective mechanism. The neuroprotective functions of NMPs in AD models are summarized in Table 3.

## 5. NMPs’ Neuroprotective Role in Huntington’s Disease

Huntington’s disease (HD) is a progressive neurodegenerative condition characterized by abnormal motor and cognitive functions. HD is triggered by mutations in the huntingtin gene (Htt) on chromosome 4, involving multiple cytosine-adenine-guanine (CAG) repeats at the exon 1 of the Htt gene [6,7,8]. Current pharmacological treatment for HD primarily utilizes atypical anti-psychotic drugs to manage hypermotor activity symptoms. However, the exact mechanisms underlying HD pathogenesis remain largely unclear, and there is currently no specific drug available to address the cognitive impairments associated with the disease [160]. Early in HD progression, research has revealed reduced CB1 mRNA and protein levels in medium spiny projection neurons of the caudate and putamen [161,162,163]. Moreover, CB1 transcription is suppressed by mutant huntingtin protein (mHtt) [164,165]. These changes in CB1 functionality significantly contribute to the cognitive, behavioral, and motor deficits observed in animal models of HD [166,167]. Consequently, there is increasing interest in pharmacological strategies to enhance CB1 signaling as a potential therapeutic approach for treating and managing HD.

### 5.1. Cannabidiol

Recent studies have evaluated the neuroprotective role of CBD in vitro using models of medium spiny projection neurons expressing mutant huntingtin protein (STHdhQ111/Q111) compared to wild type (STHdhQ7/Q7). In these models, CBD enhanced CB1 expression and GABA release and promoted CB1-independent but 5HT1A-dependent phosphorylation of CREB (p-CREB). Additionally, CBD administration in rats with 3-nitropropionic acid (3NP)-induced HD reduced CB1 receptor expression and insulin-like growth factor 1 (IGF-1) while enhancing calpain expression in striatal neurons [60]. This treatment also restored levels of GABA, substance P, and neuron-specific enolase, which are involved in generating proinflammatory markers that contribute to neuronal atrophy. Furthermore, CBD reinstated antioxidant enzyme SOD-1 and proenkephalin levels in the striatum and substantia nigra, which play crucial roles in HD pathogenesis [60]. However, despite these insights into CBD’s protective function in HD, its therapeutic utility remains controversial. While some studies indicate no improvement in animal models and human trials [168,169], others have reported beneficial effects in animal models [60,170].

**Table 4:** Neuroprotective functions of NMPs in different HD models.

Model	NMPs	Effect	Reference
STHdh(7/7) cells	CBD	ATP production, BDNF-2, and PGC1α CB1 mRNA levels ↑	[164]
Rats	CBD	mRNA SP, mRNA NSE, and mRNA SOD-2 ↑	[60]
Rats	CBD	3NP-induced GABA, Nissl-stained neurons, CB1 and IGF-1 expression, and SOD-1 expression ↓ Calpain expression ↑	[170]
RBL-2H3 cells	CBG	Human TRPV1, and rat TRPV2 ↓	[89]
HT29 cells	CBG	COX-2 enzyme, and prostaglandins ↓	[171]
Mice	CBG	Reactive microgliosis, expression of COX-2, iNOS, TNF-α, *Cd44*, and *Sgk1* ↓ PPARγ, catalase, and SOD and GSH ↑	[172]
NSC-34	CBG	*HAP1, SLC32A1, ADCY5, AKT, ATF4, DLGAP1,DRD4, GNB4, PRKCA* ↑ *ADCY9, CAMK2B, CLOCK, CREB1, DRD2, GNAL, PLD1, PPP3R1, PRKCB, SHANK1, SLC1A2, SLC18A1,* and *SLC38A1* ↓	[173]

CBD, Cannabidiol; CBG, Cannabigerol; SOD-1, Superoxide dismutase-1; SOD-2, Superoxide dismutase-2; SP, Substance P; NSE, Neuron-specific enolase; TRPV1, Transient receptor potential vanilloid 1; TRPV2, Transient receptor potential vanilloid 2; COX-2, Cyclooxygenase enzyme 2; iNOS, Inducible nitric oxide synthase; TNF-α, Tumor necrosis factor alpha; IL-6, Interleukin-6; PPARγ, peroxisome proliferator-activated receptor-γ; *cd44*, Non-kinase transmembrane glycoprotein gene; *SGK1* Serum/glucocorticoid-regulated kinase 1 gene; GSH, Glutathione; *SLC32A1*, Solute carrier family 32 member 1 gene; *ADCY5*, Adenylate cyclase 5 gene, *AKT*, Serine/threonine kinase 1 gene; *ATF4*, Activating transcription factor4 gene; *DLGAP1*, DLG-associated protein 1 gene; *DRD4*, Dopamine receptor D4 gene; G protein, Guanine nucleotide-binding protein gene; *GNAI2*, Alpha inhibiting 2 gene; *GNB4*, Beta 4 gene; *HAP1*, Huntingtin-associated protein 1 gene; *PRKCA*, Protein kinase C, alpha gene; *ADCY9*, Adenylate cyclase 9 gene; *CAMK2B*, Calcium-/calmodulin-dependent protein kinase II, beta; *CLOCK*, Circadian locomotor output cycles kaput; *CREB1*, AMP responsive element-binding protein 1; *DRD2*, Dopamine receptor D2; *GNAL*, Guanine nucleotide-binding protein, alpha stimulating, olfactory type; *PLD1*, Phospholipase; D1*PPP3R1*, Protein phosphatase 3, regulatory subunit B, alpha isoform; *PRKCB*, Protein kinase C, beta; *SHANK1*, SH3 and multiple ankyrin repeat domains 1; *SLC1A2*, Solute carrier family 1 (glial high affinity glutamate transporter), member 2; *SLC18A1*, solute carrier family 18 (vesicular monoamine), member 1; *SLC38A1*, solute carrier family 38, member 1; STHdh(7/7) cells, Cell line derived from the striatum of a mouse; RBL-2H3 cells, Basophilic leukemia cell line; HT29, Colon Cancer Line; NSC-34, Motor neuron-like cells; ↓, reduced; ↑, enhanced.

### 5.2. Δ^9^-Tetrahydrocannabivarin

Therapeutic studies of cannabinoid-based agents in HD animal models suggest that CB1 and endo vanilloid receptor agonists [174,175] and AEA reuptake inhibitors [176] can prevent hyperkinesia in the early phases of HD. This is likely due to the gradual loss of CB1 receptors [177] in the advanced stages of the disease. Although further studies are warranted, the potential use of THCV in HD is limited since it functions as an antagonist of CB1 and CB2 receptors. An improved understanding of the eCB system in HD may help identify specific NMPs or combinations that provide therapeutic or neuroprotective benefits in patients with HD.

### 5.3. Cannabidivarin

While phytocannabinoids show promise as potential treatments for motor-related diseases, no studies have evaluated the neuroprotective effects of CBDV in models of HD. Future research is needed to explore neuroprotective mechanisms within the relevant brain regions in animal models treated with CBDV.

### 5.4. Cannabigerol

CBG is a biologically active constituent of the marijuana plant, present in much smaller quantities compared to other cannabinoids. It acts as a precursor to various phytocannabinoids. CBG is a non-psychoactive compound that exhibits a wide range of biological activities [178] and is potentially a therapeutic compound for treating diseases requiring multidirectional pharmacotherapy. CBG interacts with CB1, CB2, TRPV1, and PPAR receptors [88,179,180]. Additionally, CBG suppresses the activity of FAAH, an enzyme that metabolizes anandamide (AEA), affecting its levels and biological effects. However, compared to CBD, CBG is less effective as an FAAH inhibitor [89,181]. Furthermore, CBG reduces the activity of DAGL, the enzyme responsible for the biosynthesis of 2-AG, and inhibits the activities of COX-1 and COX-2, which metabolize polyunsaturated fatty acids (PUFAs), mainly arachidonic acid, into lipid mediators [171,182,183]. CBG also acts as a neutral 5-HT1A receptor antagonist, a CB1 receptor antagonist, and a potent α2-adrenoceptor agonist [103].

CBG exhibits similar pharmacokinetic (PK) profiles in rats and mice, though it shows slower brain penetration in mice. Both species have higher concentrations of CBG following intraperitoneal (i.p.) injection compared to oral administration. However, in rats, this does not correspond to higher concentrations in brain tissue [184]. CBG has demonstrated neuroprotective effects in experimental HD models through both cannabinoid receptor-dependent and independent mechanisms. For instance, in an in vivo model of HD using 3-NP, CBG significantly reduced the expression of upregulated inflammatory markers such as COX-2, iNOS, IL-6, and TNF-α [172] and prevented the 3-NP-induced neuronal loss. Additionally, CBG reversed the activities of antioxidant enzymes, including catalase, superoxide dismutase (SOD), and glutathione (GSH), compared to control groups. CBG also rescued the expression of downregulated genes directly related to HD, such as sgk1, Cd44, and huntingtin-associated protein-1, while reducing mutant huntingtin (mHTT) protein aggregation and improving motor function [172]. Moreover, CBG was found to activate PPARγ dose-dependently in cultured striatal cells containing both wild-type and mutant huntingtin [172]. A recent transcriptomic study in motor neuron-like cells (NSC-34) revealed that CBG reduced the expression of genes involved in glutamate release, enhanced the expression of genes related to GABA release, and upregulated the dopamine D4 receptor and its downstream effectors [173], suggesting CBG’s influence on neurotransmission pathways. Future studies are needed to establish the link between these transcriptomic changes and behavioral and neuronal signaling to understand the role of CBG in neuroprotection better. The neuroprotective functions of NMPs in HD models are summarized in Table 4.

## 6. Neuroprotective Role of NMPs in Substance and Alcohol Use Disorders

Cannabinoid receptors are densely enriched in brain areas related to reward function and developing and maintaining addictive behaviors [185]. The eCB system has been implicated in the pathophysiology of addiction by modulating pathways that affect drug- and alcohol-seeking behaviors, cravings, withdrawal [24,186,187], and memory and emotional processes [188]. Alcohol [189,190] and drug abuse [191,192] act as one of the environmental factors that promote many of the neurodegenerative disorders. Therefore, studies have explored whether phytocannabinoids provide neuroprotection against substance and AUD-related disorders. In the following sections, we discuss the current evidence on the neuroprotective functions of phytocannabinoids on substance- and alcohol-use disorders.

### 6.1. Substance-Use Disorders (SUD)

In a randomized, double-blind, placebo-controlled study, CBD significantly reduced cigarette smoking, although it did not affect cravings [193]. Similarly, another study found that CBD decreased the salience and pleasantness of cigarette cues without impacting cravings, withdrawal symptoms [194], or impulsivity [195]. In an open-label crossover study involving daily users of nicotine-containing e-cigarettes, CBD was shown to reduce both the severity of nicotine withdrawal symptoms and state anxiety during e-cigarette abstinence [196]. Additionally, CBD has been found to mitigate nicotine withdrawal and hyperalgesia-inducing effects in both mice [197] and rats [198]. While the potential of other phytocannabinoids to protect against nicotine-use disorder remains unknown, future research is warranted to explore this possibility.

There has been substantial scientific discussion regarding the potential of CBD as a treatment for cannabinoid-use disorder (CUD) [199,200]. In animal models, CBD has been shown to reduce spontaneous withdrawal symptoms in THC-dependent rodents [201,202,203]. In a case study, CBD alleviated cannabis-withdrawal symptoms and improved anxiety and sleep function [203,204,205]. Additionally, CBD significantly reduced cannabis use in participants of a non-randomized open-label study [206]. Moreover, CBD mitigated adverse cognitive and mental health effects [201,202] in long-term THC users, including reduced cognitive deficits, psychotic-like and depressive symptoms, and increased hippocampal volumes [207] and resting-state functional connectivity [208]. These studies suggest that the antipsychotic and anxiolytic properties of CBD may improve psychological and cognitive functions related to long-term cannabis use—the use of other NMPs to combat the effects of THC warrants further study.

CBD has been studied in opioid-use disorders and has been shown to mitigate morphine reward and reduce the reinstatement of heroin-seeking behavior in rats [209,210], as well as alleviate opioid withdrawal symptoms in rodents [211,212]. It also decreased naloxone-precipitated jumping behavior in mice [213]. CBD prevented the reinstatement of oxycodone-induced conditioned place preference (CPP) and rescued recognition memory deficits in adolescent male mice. These mice exhibited increased CB1 receptors and a reduced mu-opioid receptor (MOR) expression in the prefrontal cortex (PFC) [214]. CBG was found to reverse oxaliplatin-associated mechanical sensitivity [215] and attenuate the acute antinociceptive effects of morphine through interactions with α2-adrenergic, CB1, or CB2 receptors [213]. These findings suggest that continued research on CBD, CBG, and other NMPs could enhance our understanding of their potential in treating pain and substance use disorders. The neuroprotective functions of NMPs in SUD models are summarized in Table 5.

### 6.2. Alcohol-Use Disorders (AUD)

CBD has shown promise in preventing alcohol abuse and mitigating alcohol-related effects, owing to its anxiolytic properties [216,217]. Preclinical studies strongly suggest that the eCB system plays a significant role in the motivational properties of alcohol and its effects [24,218,219,220]. Specifically, antagonism of the CB1 receptor has been shown to suppress rodent alcohol consumption [24,218,219,220,221]. As CBD may act as a negative allosteric modulator of the CB1 receptor, this supports the notion that CBD could be a promising treatment for AUD.

CBD has demonstrated protective effects against alcohol-induced neurodegeneration. When administered alongside binge ethanol exposure, CBD rescued neurodegeneration in the hippocampus and entorhinal cortex [222]. Transdermal administration of CBD also significantly reduced neurodegeneration in the entorhinal cortex [223]. Furthermore, CBD has been shown to prevent alcohol-induced cell death and improve neuronal viability in rat hippocampal cultures [224]. In alcohol-dependence models, CBD inhibited impulsive choice behavior [225,226], a factor linked to both alcohol use and the risk of relapse [227,228]. CBD has also been found to reduce alcohol consumption [203], motivation, and relapse in a two-bottle choice paradigm [229]. In these studies, CBD reduced ethanol-induced hypothermia and handling-induced convulsions without affecting blood ethanol concentration [229]. Moreover, CBD does not alter breath alcohol levels in humans [230]. CBD also reversed alcohol-induced changes in gene expression, such as tyrosine hydroxylase in the ventral tegmental area, Oprm1, CNR1, and GPR55 in the nucleus accumbens (NAcc) while significantly enhancing CB2 receptor expression in the Nacc [229].

In selectively bred Sardinian alcohol-preferring (sP) rats, CBD significantly reduced lever responses for alcohol and the amount of self-administered alcohol [231]. Although the exact neuroprotective mechanisms of CBD require further investigation, these findings strongly suggest that CBD may be beneficial for treating AUDs. In another study, CBD significantly reduced alcohol-withdrawal symptoms such as anxiety behavior and altered gene expression in related brain regions [232]. In the alcohol binge-like drinking model, CBD inhibited alcohol consumption and preference, normalized abnormal corticolimbic calcitonin gene-related peptide (CGRP) expression, and reduced reward and aversion-related hyper-responsivity and glucocorticoid levels in rats [233]. CBD also significantly mitigated the blunted stress response and corticosterone levels caused by binge-like alcohol exposure, restored dopamine transmission, and facilitated excitatory postsynaptic strength and remodeling in rats [234]. Additionally, CBD inhibited the development of tolerance to alcohol’s hypothermic and sedative effects and restored reduced CB2R gene transcription in the striatum caused by ethanol [235]. In another alcohol-withdrawal study, CBD reduced anxiety-like behaviors in mice [236]. However, some studies show limitations: CBD failed to attenuate alcohol-induced locomotor sensitization [237], and oral CBD did not prevent alcohol seeking, self-administration, or intake in baboons [238]. Although the precise mechanisms remain unknown, these studies indicate that CBD and potentially other phytocannabinoids could offer neuroprotection against alcohol toxicity and AUD.

Fetal Alcohol Spectrum Disorder (FASD) embodies a range of neurobehavioral impairments caused by prenatal and postnatal exposure to ethanol. Recent studies have shown that CBD treatment during the peri-adolescence period can mitigate the increased levels of TNFα and IL-6 in the hippocampus, as well as the cognitive deficits associated with prenatal and postnatal alcohol exposure [239]. Data from four case studies suggest that CBD has neuroprotective effects, reducing FASD symptoms such as restlessness, aggression, agitation, impulsivity, and high scores on the Nisonger Disruptive Behavior Scale [240]. Additionally, CBD administration has been shown to reverse alcohol-induced gene-expression defects, anxiety, depressive-like behaviors, and cognitive impairments in animal models exposed to alcohol both prenatally and postnatally [241]. Although the precise mechanisms are not yet fully understood, early research suggests that CBD, or possibly other phytocannabinoids, could provide neuroprotective effects against the developmental impact of alcohol exposure. The neuroprotective functions of NMPs in AUD models are summarized in Table 6.

## 7. Conclusions

In summary, the therapeutic potential of cannabis sativa extends well beyond the widely studied CBD, encompassing a diverse range of lesser-known phytocannabinoids that show promise in addressing various neurological disorders. The neuroprotective functions of these NMPs, particularly their antioxidant, anti-inflammatory, and immune-modulating properties, offer new avenues for research and treatment. While the pharmacological mechanisms of many NMPs remain underexplored, emerging studies suggest their potential to develop novel therapies for brain disorders. As research continues to unfold, these findings could pave the way for innovative cannabinoid plant-based treatments that go beyond the scope of traditional approaches, offering new hope in neuroprotection and disease management.

## Figures and Tables

**Table 1:** Neuroprotective functions of NMPs in epilepsy models.

Model	NMPs	Effect	Reference
Pilocarpine–epilepticus rat	CBD	Convulsant ↓ Neurodegenration ↓	[49]
PTZ seizures	CBD and CBG	Nav current in cells ↓	[104]
Epilepsy-spontaneous LFPs in cells	CBDV	Amplitude and duration of LFPs ↓ Mg^2+^ free induced LFPs frequency ↑	[93]
Epilepsy in transfected cells (TRPV1, TRPV2, and TRPA1)	CBDV + CBD	Convulsant ↓ Phosphorylation of TRPV1 at the S800 site ↑	[108]
Electrophysiology (epileptiform bursting) (in vitro)	Δ^9^-THCV	Epileptiform burst ↓	[81]
PTZ seizures	CBDV	Seizure severity ↓ Latency to first signs of seizure ↑	[93,97,98]
PTZ seizures	Δ^9^-THCV	Median seizure severity, duration, progression, or latency was unaffected	[81]
6-hydroxytryptamine or LPS in rats and mice	Δ^9^-THCV	Neuronal loss, microglial activation, ↓ TH positive neurons and Motor activity↑	[109]
Rat model	CBD	Convulsant ↓ Seizure severity ↓	[61]
Rat (GEPR-3) strain	CBD	Seizure ↓	[67]

CBD, Cannabidiol; CBDV, Cannabidivarin; CBG, Cannabigerol; ∆^9^-THCV, ∆^9^-tetrahydrocannabivarin; LFS, local field potentials; PTZ, pentylenetetrazole; LPS, lipopolysaccharide; TRPV, transient receptor potential vanilloid; TH, Tyrosine hydroxylase; GEPR-3, genetically epilepsy-prone rat; ↓, reduced; ↑, enhanced.

**Table 5:** Neuroprotective functions of NMPs in different SUD models.

Model	NMPs	Effect	Reference
Humans	CBD (+THC)	Anxiety ↓	[199]
Humans	CBD (+THC)	Satiety ↑	[200]
Humans	CBD	Emotion, reward processing, And effects of THC ↓	[202]
Human	CBD	Withdrawal, anxiety, and dissociative symptoms ↓	[204]
Human	CBD	Anxiety and cannabis use ↓ Sleep ↑	[205]
Humans	CBD	Cannabis use ↓	[206]
Humans	CBD	Psychological symptoms ↓ Cognition ↑	[207]
Human	CBD	Functional connectivity ↑	[208]
Wistar rats	CBD	Morphine-reward behavior ↓	[209]
Rat	CBD	Heroin-seeking behavior, CB_1_R expression ↓	[210]
Mice	CBD	Anxiety, *Cnr1*, and *Pomc* ↓ Motor activity and TH expression, ↑	[211]
Mice	CBD	Gastrointestinal symptoms and jumping behavior ↓	[212]
Mice	CBD	Mechanical sensitivity and jumping behavior ↓	[213]
Rats	CBD	Locomotor hyperactivity and MOR ↓ Recognition memory and CB1R expression ↑	[214]
Mice	CBG	Mechanical sensitivity, *Aif1*, *Ccl2*, *Calca*, and *Tlr4* ↓	[215]

CBD, Cannabidiol; THC, Tetrahydrocannabinol; CBG, Cannabigerol; TH, tyrosine hydroxylase; Pomc, Proopiomelanocortin; Cnr1, Cannabinoid receptor; MOR Mu-opioid receptor; *Aif1*, Allograft Inflammatory Factor 1; *Ccl2*, C-C Motif Chemokine Ligand 2; *Calca*, Calcitonin Related Polypeptide Alpha; Tlr4, Toll-Like Receptor 4; ↓, reduced; ↑, enhanced.

**Table 6:** Neuroprotective functions of NMPs in different AUD models.

Model	NMPs	Effect	Reference
Rat	CBD	Anxiolytic effect ↑	[216]
Rats	CBD	Social interaction ↑	[217]
Rats	CBD	Neurodegeneration ↓	[218]
Rats	CBD	Neurodegeneration ↓	[223]
Hippocampal cultures	CBD	Neuroprotection ↑	[224]
Mice	CBD	Alcohol intake, TH, Oprm1, and CB_1_R and GPR55 gene expression ↓	[225]
Humans	CBD	BrAC ↓	[230]
sP Rats	CBD	Lever responses to self-administered alcohol ↓	[231]
Mice	CBD (+THC)	Locomotor sensitization ↓	[237]
Baboons	CBD	Alcohol seeking, self-administration, and drinking patterns ↓	[238]
Mice (SAW model)	CBD	Rearings, groomings, anxiogenic behavior, *Cnr2,* and *Opmr1* expression ↑ *Th* and *Pomc* gene expressions ↓	[232]
Rats	CBD	CGRP, alcohol consumption, and preference ↓	[233]
Rats	CBD	Corticosterone ↓ DA and postsynaptic strength ↑	[234]
Rats	CBD	Hypothermic and sedation CB1R, DRD1, and DRD2 mRNA ↓ CB2R gene transcription ↑	[235]
Mice	CBD	Anxiety behavior, S100β, and Iba1 ↓	[236]
Mice	CBD	Cognitive deficits and TNFα IL-6 ↑	[239]
Human	CBD	Disruptive behavior score ↓	[240]
Mice	CBD	Emotional cognitive disturbance ↓	[241]

CBD, Cannabidiol; THC, Tetrahydrocannabinol; Oprm1, Opioid Receptor Mu 1; GPR55, G Protein-Coupled Receptor 55; CB_1_R, Cannabinoid Receptor 1, TH, Tyrosine hydroxylase; BrAC, Breath alcohol level; sP, Sardinian alcohol-preferring; *Cnr2*, Cannabinoid Receptor 2 gene; SAW, Spontaneous alcohol withdrawal; CGRP, Calcitonin gene-related peptide; DA, Dopamine; DRD1, Dopamine receptor D1; DRD2, Dopamine receptor D2; S100B, calcium-binding protein; Iba, Ionized calcium-binding adaptor molecule 1; TNF-α, Tumor necrosis factor-alpha; IL-6, Interleukin-6, SAW, Spontaneous alcohol withdrawal; ↓, reduced; ↑, enhanced.
